# Phosphoproteomics-based peptide ligand-receptor kinase pairing. Commentary on: “A peptide hormone and its receptor protein kinase regulate plant cell expansion”

**DOI:** 10.3389/fpls.2015.00224

**Published:** 2015-04-09

**Authors:** Elisabeth Stes, Kris Gevaert, Ive De Smet

**Affiliations:** ^1^Department of Plant Systems Biology, Vlaams Instituut voor BiotechnologieGhent, Belgium; ^2^Department of Plant Biotechnology and Genetics, Ghent UniversityGhent, Belgium; ^3^Department of Medical Protein Research, Vlaams Instituut voor BiotechnologieGhent, Belgium; ^4^Department of Biochemistry, Ghent UniversityGhent, Belgium

**Keywords:** peptide ligand, receptor kinase, phosphorylation, mass spectrometry, phosphoproteomics

Small signaling peptides and their receptors play essential roles in plant growth and development (Murphy et al., 2012; Czyzewicz et al., 2013; Matsubayashi, 2014). *Arabidopsis thaliana* encodes over 600 putative receptor-like kinases and more than 1000 potential secreted peptides (Shiu and Bleecker, 2001; Lease and Walker, 2006), and similar numbers can be expected in other plant species (Shiu et al., 2004; Lehti-Shiu et al., 2009). Taking into account that one peptide can bind or signal through multiple receptors and one receptor can recognize several peptides (Ogawa et al., 2008; Kinoshita et al., 2010; Lee et al., 2011; Shinohara et al., 2012), there is an enormous number of possible peptide ligand-receptor kinase pairs. However, this number of possibilities is in stark contrast with the very few peptide ligand-receptor kinase pairs that have been identified (Butenko et al., 2009; Murphy et al., 2012; Czyzewicz et al., 2013; Endo et al., 2014). Evidently, unambiguous identification of a ligand is crucial to fully understand receptor kinase–mediated signaling pathways (Hirakawa et al., 2008; Jia et al., 2008; Ogawa et al., 2008; Lee et al., 2012; Uchida et al., 2012; Okamoto et al., 2013; Tabata et al., 2014).

The identification of ligand-receptor pairs is technically very challenging, as the genes encoding them regularly belong to gene families with multiple members and are often low expressed, and this only in certain cell types or during specific developmental stages. Therefore, various strategies were followed to identify candidate pairs, such as transcriptional analyses at cellular resolution, microscopic characterization of loss and gain-of-function plants, genetic interaction studies, and biochemical assays demonstrating direct physical interactions (see e.g., Murphy et al., 2012 and Czyzewicz et al., 2013 for more details).

To study the physical interaction of ligands with their receptors, Butenko et al. (2014) recently developed a rapid cellular bioassay that uses the oxidative burst response in *Nicotiana benthamiana* leaves as readout for activation of (ectopically expressed) receptors by synthetic peptides. However, while a broad range of receptor kinases might be able to activate an oxidative burst, it is likely that this approach cannot be applied to all peptide ligands and/or receptor kinases. In addition, prior knowledge on potential receptor candidates facilitates such studies and there is the requirement for expressing the receptor in *N. benthamiana*. A similar approach relies on chimeric receptors and monitoring of luciferase activity of known targets in protoplasts transiently expressing signaling components (Albert et al., 2010; Mueller et al., 2012).

Another approach was used by Tabata et al. (2014). Here, overexpression of individual *A. thaliana* leucine-rich repeat receptor kinases (LRR-RKs) from subfamilies X and XI in tobacco BY-2 cells was combined with photoaffinity labeling of these LRR-RKs by a biologically active small signaling peptide analog derivatized with ^125^I-labeled photoreactive 4-azidosalicylic acid ([^125^]ASA). This revealed that two related LRR-RKs of subfamily XI directly and specifically interact with C-TERMINALLY ENCODED PEPTIDE 1 (CEP1). Subsequently, this interaction was confirmed by demonstrating that *cepr1 cepr2*, a double loss-of-function mutant in the identified CEP1 receptor genes, was insensitive to synthetic CEP1 peptide in a root growth assay. This novel approach represents a major leap forward regarding ligand–receptor pairing, but does not take the ligand-receptor interactions into account that rely on a protein complex status involving co-receptors and/or interacting proteins.

In this General Commentary, we would like to highlight an original proteomics-driven approach overcoming the above-described limitations, including the need for constructs, which was employed by the Sussman lab in their quest for the receptor of the secreted RAPID ALKALINIZATION FACTOR (RALF) peptide (Haruta et al., 2014) (Figure 1). Specifically, the phosphorylation status of plasma membrane proteins—in their natural *in planta* environment—was studied in response to treatment with recombinant, biologically active RALF peptide. To obtain quantitative phosphoprotein profiles, ^15^N metabolic labeling of *A. thaliana* seedlings was combined with mass spectrometry–based phosphoproteomics. This strategy allowed the identification of five plasma membrane proteins that displayed a RALF-induced change in phosphorylation pattern, of which two receptor kinases. These can be considered as putative RALF receptors, given that ligand binding likely results in an immediate change in phosphostatus, as illustrated through phosphorylation of the brassinosteroid receptor BRI1 upon ligand binding (Wang et al., 2005). Among these was the FERONIA (FER) receptor kinase, which, following necessary biochemical and functional characterization, was indeed confirmed as a RALF receptor. It is hypothesized that, upon recognition of the peptide ligand, phosphorylation at the C-terminus activates the kinase and initiates a RALF-induced signaling cascade. Additionally, the phosphoproteome analysis pinpointed a plasma membrane H^+^-ATPase, AHA2, as a potential downstream protein and/or putative FER substrate. Their findings support a model where RALF recognition by the FER kinase affects proton transport, and consequently cell elongation and plant development (see also Murphy and De Smet, 2014). This powerful and straightforward approach hence identified RALF and FER as peptide–receptor pair and shed light on the molecular mechanism that regulates cell elongation. However, it should be taken into account that depending on the experimental set-up, the selected treatments, and the developmental and physiological growth stage, this approach might not give the complete picture. This is exemplified by the *fer4* knockout mutant, which is not completely insensitive to RALF at higher concentrations, suggesting there are likely other RALF receptors. Nevertheless, this is a powerful approach that allows identifying receptors for selected ligands and/or universal downstream responses, without (transient) expression of components.

**Figure 1:**
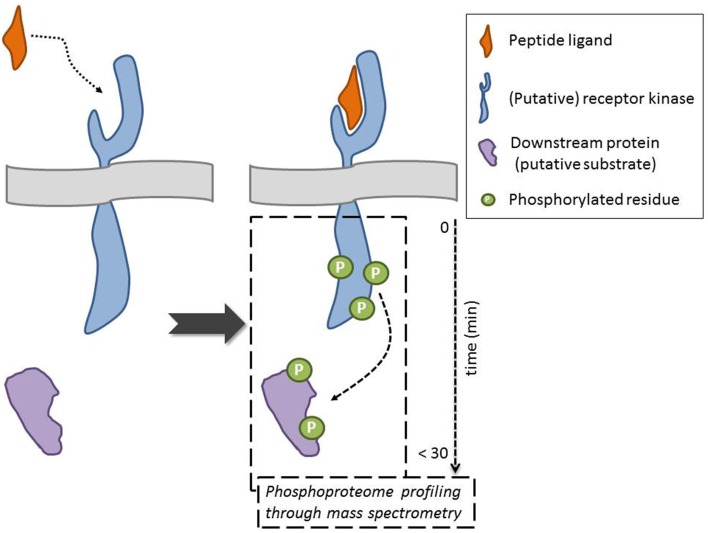
**Mass spectrometry-based approaches point to putative receptor kinase for orphan ligand and to downstream components and/or potential substrates following ligand binding through evaluation of changes in the phosphorylation status of proteins**. The ideal scenario, where indeed the peptide interacts with the receptor, is depicted (dotted arrow). An alternative outcome is, for example, that the peptide indirectly effects the phosphorylation status of a membrane-associated receptor kinase. The phosphorylation of the downstream protein can be direct (receptor kinase substrate) or indirect (requiring intermediate kinases) (dashed arrow).

In conclusion, enormous progress has been made in the matching of peptide ligand–receptor kinase pairs in the last years, with mass spectrometry-driven phosphoprotein profiling as a promising strategy that can be applied to identify receptors for orphan ligands and to progressively close the gap in the plant peptide–receptor field.

## Conflict of interest statement

The authors declare that the research was conducted in the absence of any commercial or financial relationships that could be construed as a potential conflict of interest.
